# Combining vascular and nerve fiber layer thickness measurements to model glaucomatous focal visual field loss

**DOI:** 10.1111/nyas.14732

**Published:** 2022-01-14

**Authors:** Martin Kallab, Nikolaus Hommer, Andreas Schlatter, Jacqueline Chua, Bingyao Tan, Doreen Schmidl, Cornelia Hirn, Oliver Findl, Leopold Schmetterer, Gerhard Garhöfer, Damon Wong

**Affiliations:** ^1^ Department of Clinical Pharmacology Medical University of Vienna Vienna Austria; ^2^ Vienna Institute for Research in Ocular Surgery (VIROS) Hanusch Hospital Vienna Austria; ^3^ Singapore Eye Research Institute Singapore National Eye Centre Singapore Singapore; ^4^ Academic Clinical Program Duke‐NUS Medical School Singapore Singapore; ^5^ SERI‐NTU Advanced Ocular Engineering (STANCE) Singapore Singapore; ^6^ School of Chemical and Biomedical Engineering Nanyang Technological University Singapore Singapore; ^7^ Department of Ophthalmology Hanusch Hospital Vienna Austria; ^8^ Institute of Molecular and Clinical Ophthalmology Basel Basel Switzerland; ^9^ Center for Medical Physics and Biomedical Engineering Medical University of Vienna Vienna Austria

**Keywords:** glaucoma, optical coherence tomography angiography, retinal nerve fiber layer, visual field, structure–function correlation

## Abstract

We compare the focal structure–function correlation of structural measurements of peripapillary retinal nerve fiber layer thickness (RNFL‐T) using optical coherence tomography (OCT), capillary density (CD) measurements using OCT‐angiography (OCT‐A), or a combination of both, with visual field deviation (VFD) in early to advanced glaucoma. Primary open angle glaucoma patients (*n* = 46, mean ± SD age: 67 ± 10 years; VF mean deviation: −10.41 ± 6.76 dB) were included in this cross‐sectional study. We performed 30–2 standard automated perimetry OCT (3.5‐mm diameter ring scan) and 15°×15° OCT‐A (superficial vascular complex slab). Based on a nerve fiber trajectory model, each VF test spot was assigned to an OCT‐A wedge and an OCT ring‐sector. Two univariate linear models (*M_v_* and *M_t_*) using either CD‐based vascular (*M_v_*) or RNFL‐T–based thickness information (*M_t_*) and one multivariate model using both (*M_v:t_*) were compared in their associations with measured focal VFD, which were higher for the multivariate model *M_v:t_* (mean ± SD correlation coefficient: 0.710 ± 0.086) than for either nested model (0.627 ± 0.078 for *M_v_* and 0.578 ± 0.095 for *M_t_*). Using a focal visual field approach, the combination of RNFL‐T and CD showed better structure–function correlations than thickness or vascular information only.

## Graphical abstract

Early diagnosis and adequate progression monitoring of primary open angle glaucoma (POAG) requires assessment of structural and functional parameters. We present focal structure–function correlations for capillary density (CD) and retinal nerve fiber layer thickness (RNFL‐T) with visual field deviation, using both univariate and multivariate modeling where the combined use of CD and RNFL‐T was identified to have overall advantageous properties over vascular or thickness measurements alone, with focal exceptions for the former.

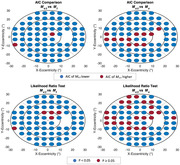

## Introduction

Early diagnosis and adequate progression monitoring of primary open angle glaucoma (POAG) requires the assessment of structural and functional parameters.^1^, ^2^ Retinal nerve fiber layer thickness (RNFL‐T) measured by means of peripapillary optical coherence tomography (OCT) is currently the most commonly used structural parameter, while standard automated perimetry (SAP) supplies clinicians with information on the associated visual field deviation (VFD), which is the most commonly used functional parameter. However, the structure–function (SF) correlation between RNFL‐T and VFD has been found to be relatively weak, in particular in later stages of disease, when VFD further progresses despite seemingly stable or only slowly progressing RNFL‐T thinning.^3^ This so‐called “floor effect” of the RNFL has been extensively studied in glaucoma^4^, ^5^, ^6^, ^7^ and is commonly explained by persistence of the non‐axon proportion (i.e., blood vessels and glial cells) of the RNFL.^5^

Since the emergence of optical coherence tomography angiography (OCT‐A), the applicability of capillary density (CD) for glaucoma diagnostics has been intensively evaluated.^8^ Indeed, peripapillary or perifoveal CD have been proposed as more sensitive alternatives to RNFL‐T, and various studies found evidence for favorable SF correlations for peripapillary CD^9^, ^10^, ^11^, ^12^, ^13^, ^14^, ^15^, ^16^, ^17^, ^18^, ^19^, ^20^, ^21^, ^22^, ^23^ or perifoveal CD^13^, ^17^, ^20^, ^21^, ^24^ with SAP as compared to RNFL‐T, partly attributing this superiority to lacking or less pronounced flooring, especially in later glaucoma.

However, both global and sectoral analyses fail to provide focal information on correlation, while results from studies in early glaucoma only may be limited in their transferability to more advanced stages. Thus, we applied a recently introduced focal approach,^23^ based on a nerve fiber trajectory model developed by Jansonius and coworkers,^25^, ^26^ to a dataset of early to advanced POAG with the aim to focally model the SF correlation of CD, RNFL‐T, and VF loss using OCT‐A, structural OCT, and SAP, respectively.

## Materials and methods

### Study subjects

All included subjects participated in an ocular imaging study (ClinicalTrials.gov identifier: NCT03870230) assessing functional, structural, and blood flow–related parameters in glaucoma patients and were recruited between April 2019 and January 2021. This study was conducted in compliance with the Declaration of Helsinki and the Good Clinical Practice guidelines of the European Union and was approved by the Ethics Committee of the Medical University of Vienna. Informed consent was obtained from all subjects before any study‐related procedures were performed.

### Experimental paradigm

The following examinations were performed in all subjects to assess eligibility: patient medical history and concomitant medication, pregnancy testing in women with childbearing potential, best‐corrected visual acuity, visual field (VF) testing using SAP, and slit‐lamp examination, including dilated funduscopy and applanation tonometry. The inclusion criteria were diagnosis of POAG with glaucomatous optic neuropathy and VF loss compatible with glaucoma. The exclusion criteria were current smoking, exfoliation glaucoma, pigmentary glaucoma or any other secondary cause for glaucoma, history of acute angle closure or angle closure glaucoma, intraocular surgery within 6 months, diabetes, untreated hypertension (SBP >160 mmHg or DBP >95 mmHg), and pregnancy.

After inclusion, peripapillary OCT‐A volume and circumpapillary OCT scans were performed in one eye per subject.

### Measurements

#### SAP

A 30–2 VF test using the SITA‐Standard protocol was performed with a Humphrey perimeter (Zeiss, Dublin, Ireland). Only VF tests with sufficient compliance parameters were processed (fixation losses: <33%, false‐positive/false‐negative errors <20%). Analysis protocols of SAP were exported and focal VF deviations (*fVFD*) of all test locations were extracted and tabulated.

#### Peripapillary OCT‐A scans

**Figure 1:**
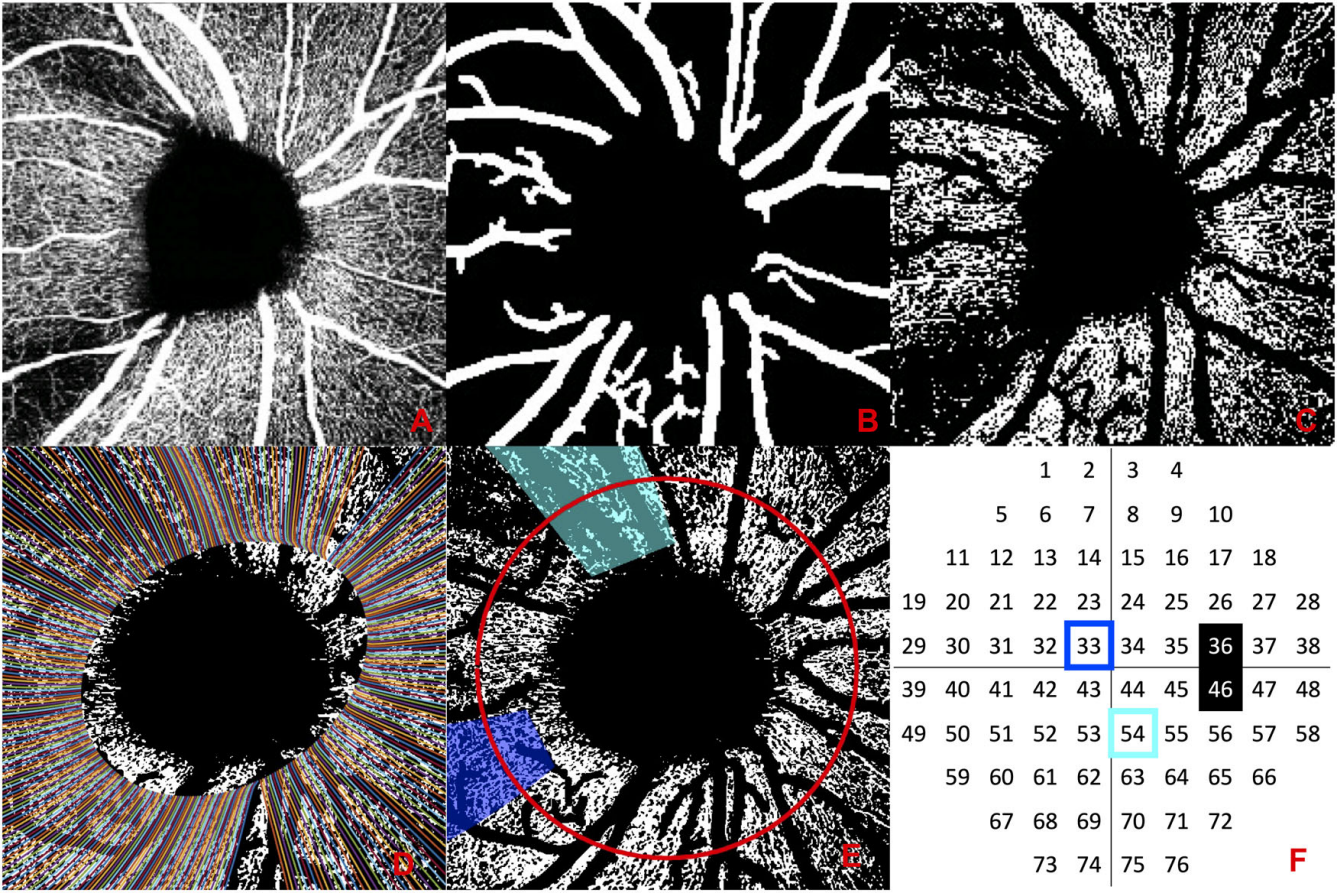
OCT‐A scan processing and nerve fiber trajectory model application. (A) Raw OCT‐A image as exported from the device. (B) Large vessel mask. (C) Binarized OCT‐A image after large vessel removal. (D) Nerve fiber trajectories were fitted to the individual fovea–disc distance and fovea–disc angle. (E) 95% CI peripapillary wedges (light‐blue and dark‐blue) were applied to the OCT‐A image and the structural OCT (red circle). (F) Visual field locations 33 and 54 corresponding to the dark‐blue and light‐blue peripapillary wedges are highlighted accordingly; visual field locations 36 and 46 corresponding to the blind spot are highlighted in black.

15°×15° OCT‐A volume scans (384 B‐Scans and 384 A‐Scans/B‐Scan) were acquired using the standard OCT‐A algorithm (full spectrum amplitude decorrelation) of the Heidelberg Spectralis OCT (Heidelberg Engineering, Heidelberg, Germany). Only scans with sufficient quality were used for analysis. Superficial vascular complex slabs as generated by the built‐in segmentation software of the OCT‐A device were exported to MATLAB (MathWorks, Natick, MA) and each image was processed using a previously described customized algorithm (Fig. 1A–C).^23^ In short, capillaries were binarized before large vessels were isolated using a Hessian‐based Frangi‐filter^27^ protocol. Finally, all images were rescaled and cropped to 4.11 mm/960 pixels to adjust for differing focus settings due to individual patient refraction.

#### Peripapillary OCT scans

We acquired 3.5‐mm diameter structural OCT ring scans using the glaucoma module of the Heidelberg Spectralis OCT (Heidelberg Engineering). Circumpapillary RNFL‐T values at the 3.5‐mm diameter ring position (focus‐adjusted by the device) were extracted using the automatic RNFL‐segmentation of the Heidelberg glaucoma module, which was manually controlled for segmentation errors.

### Nerve fiber trajectory model setup and analysis

As previously described in Wong *et al*.,^23^ the Jansonius nerve fiber trajectory model^25^, ^26^ can be used to match VF locations with the corresponding wedges in a peripapillary OCT‐A scan and the corresponding circular segment in a circumpapillary OCT scan.

In short, information on the fovea–disc distance and the fovea–disc angle were extracted from the SLO image acquired together with the circumpapillary OCT. Based on these two parameters, the trajectory model was fitted to the OCT‐A scan and the structural OCT scan of every patient (Fig. 1D and E). For analysis, each VF location was linked to a range of (95% CI) corresponding nerve fiber trajectories, which defined the region of interest (ROI) on the OCT‐A for focal CD (*fCD*) calculation as the proportion of white pixels ( = perfusion) in the ROI (Fig. 1E and F). The mean thickness of the RNFL in the OCT ring scan segment covered by the ROI was defined as the focal RNFL‐T (*fRNFL‐T*) (Fig. 1E and F). All processing was performed in MATLAB.

### Statistical analysis

Associations of *fVFD* with *fCD* and *fRNFL‐T*, representing vascular (v) and thickness (t) information, were modeled for every VF location (*n* = 73) using two univariate regression models (*M_v_* and *M_t_*) with *fCD* (*M_v_*, Eq. 1) or *fRNFL‐T* (*M_t_*, Eq. 2) as independent variables and one multivariate regression model (*M_v:t_*, Eq. 3), with both *fCD* and *fRNFL‐T* as independent variables, including an interaction term. The regression equations were as follows:

(1) $$\left(fVFD\right)=\beta_{0,M_{v}}+\beta_{1,M_{v}}\left(fCD\right)+\varepsilon$$

(2) $$\left(fVFD\right)=\beta_{0,M_{t}}+\beta_{1,M_{t}}\left(fRNFL-T\right)+\varepsilon$$

(3) $$\begin{array}{ccc} \left(fVFD\right) & = & \beta_{0,M_{v:t}}+\beta_{1,M_{v:t}}\left(fCD\right)\\ & & +\;\beta_{\text{2,}M_{v:t}}\left(fRNFL-T\right)\\ & & +\;\beta_{\text{3,}M_{v:t}}\left(fCD\right)\left(fRNFL-T\right)+\varepsilon \end{array}$$

Pearson correlation coefficients (*r*_p_) were used to compare the associations with measured *fVFD*s. Linear models were compared using the Akaike information criterion (AIC) and likelihood‐ratio tests.

To mathematically describe the breakpoint and corresponding slopes (point estimates and 95% CI), segmented regression analysis on a linear mixed model, in which the focal parameters were used as the fixed effect and the random effects were used to account for multiple VF data from the same individual, was performed. Separate models were evaluated for *fRNFL‐T* and *fCD*. The Davies test was used to test for the existence of a significant change in the slope (breakpoint) of the relationship between *fVFD* and *fCD* or *fRNFL‐T*.

Global SF relationships were also evaluated using Eqs. ((1), (2), (3)) for global structural parameters (RNFL‐T and CD) and global VFD, with Pearson correlation coefficients used to assess the strength of the associations.

Stata 13.1 (StataCorp, College Station, TX) and R (R Foundation for Statistical Computing, Vienna, Austria), comprising the package “segmented,” were used for statistical analysis. *P* values below 0.05 were treated as statistically significant.

## Results

### Study population

Fifty‐one POAG patients were enrolled for the present study. After we excluded one patient due to high myopia (>6 dpt) and four patients due to poor OCT‐A acquisition quality, 46 patients remained. Regarding their VFD, POAG patients exhibited the following characteristics: mean ± SD: −10.41 ± 6.76 dB, range (min./max.): −25.7/0.4 dB, median: −9.1 dB, and interquartile range: 10.6 dB. According to a VF loss classification system proposed by the 5th Guidelines of the European Glaucoma Society,^28^ 17 patients were classified as advanced, 14 as moderate, and 15 as early glaucomatous VF loss. All baseline characteristics are summarized in Table 1. The number of prescribed IOP‐lowering medications and type of glaucoma surgeries are shown in Table 2.

**Table 1:** Summary of baseline characteristics

Baseline characteristics (unit)	Mean±SD or number
*n*	46
Age (years)	67 ± 10
Gender (female/male)	28/18
SBP (mmHg)	135 ± 14
DBP (mmHg)	77 ± 10
MAP (mmHg)	104 ± 12
IOP (mmHg, study eye)	14 ± 3
OPP (mmHg, study eye)	55 ± 8
Time since glaucoma diagnosis (years)	9 ± 7
VF MD (dB)	−10.41 ± 6.76
Cup‐disc ratio	0.9 ± 0.1
Global capillary density (%)	0.18 ± 0.08
Global nerve fiber layer thickness (μm)	62 ± 13

DBP, diastolic blood pressure; IOP, intraocular pressure; MAP, mean arterial pressure; OPP, ocular perfusion pressure; SBP, systolic blood pressure; VF MD, visual field mean deviation.

**Table 2:** Number of prescribed IOP‐lowering medications and type of surgeries

Number of medications	No surgery	Trabeculectomy	MIGS	**Total**
0	2	8	1	**11**
1	5	0	1	**6**
2	6	0	1	**7**
3	16	3	0	**19**
4	3	0	0	**3**
**Total**	**32**	**11**	**3**	**46**

MIGS, minimally invasive glaucoma surgery.

### Global analysis

We found a significant correlation between global structural parameters (RNFL‐T, CD, RNFL‐T and CD) with global VFD (*P* < 0.001 for all three), with an *r*_p_ of 0.536 (RNFL‐T), 0.749 (CD), and 0.772 (RNFL‐T and CD), respectively.

### Focal analysis‐model comparison

**Figure 2:**
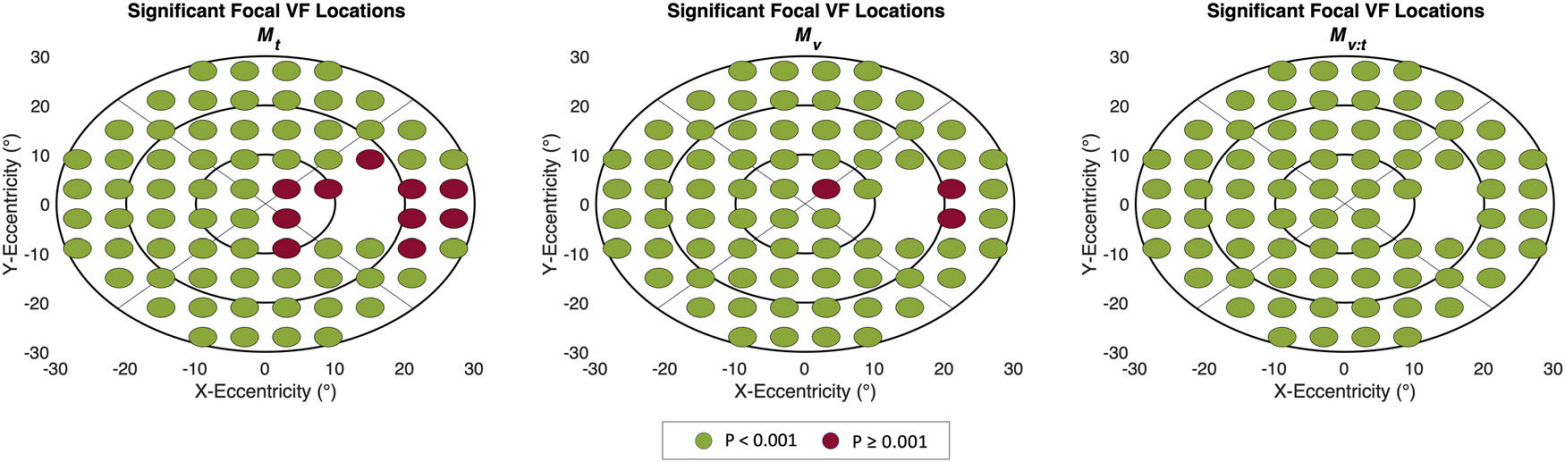
Significant (green) and not significant (red) focal VF locations at a significance level of 0.001 for the three compared models *M_t_* (left) *M_v_* (middle), and *M_v:t_* (right). *x* and *y* axes scaling corresponds to eccentricity from fixation in degrees.

We analyzed the associations of *M_v_*, *M_t_*, and *M_v:t_* with *fVFD*. *M_v:t_* resulted in a mean *r*_p_ of 0.710 ± 0.086 with measured *fVFD*, while *M_v_* and *M_t_* showed an *r*_p_ of 0.627 ± 0.078 and 0.578 ± 0.095, respectively. Correlations were significant at a *P* < 0.05 significance level for all VF locations and all three models. We next lowered the significance level to *P* < 0.001, which resulted in a loss of significance in 10 of the 73 VF locations (14%) in *M_t_* and three VF locations (4%) in *M_v_*. All VF locations remained significant in *M_v:t_* (Fig. 2).

**Figure 3:**
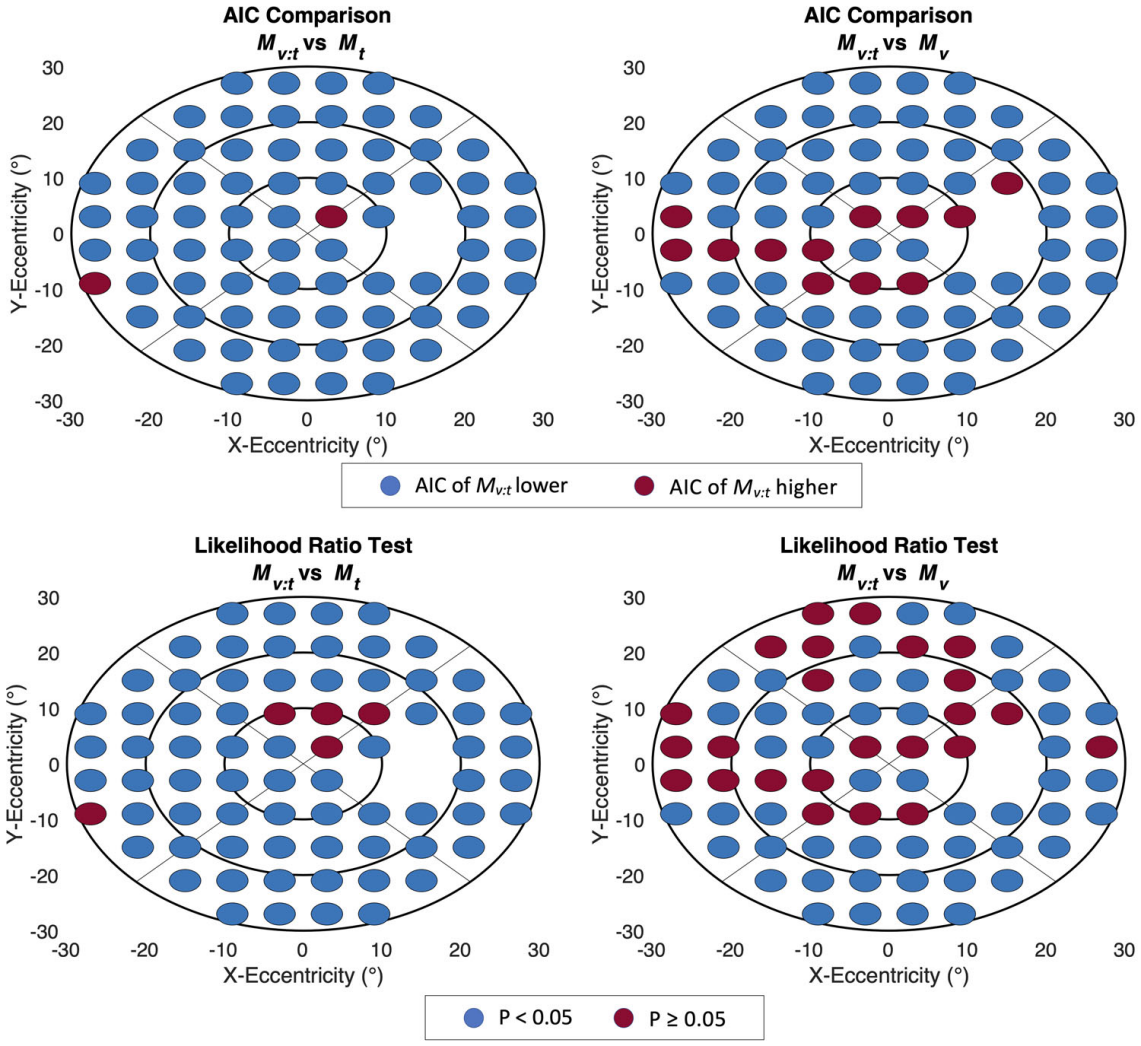
Performance of *M_v:t_* as compared to *M_t_* or *M_v_* at every VF test location based on AIC or likelihood‐ratio tests. Blue = *M_v:t_* AIC lower/likelihood ratio test significant; red = *M_v:t_* AIC higher/likelihood ratio test not significant. *x* and *y* axes scaling corresponds to eccentricity from fixation in degrees.

For a further comparison of the models, the AIC was calculated for each regression model at all VF locations, suggesting the superiority of *M_v:t_* over *M_t_* in 71 (97%) and over *M_v_* in 61 (84%) VF locations. Visual representation of the latter comparison revealed that VF locations showing no AIC drop for *M_v:t_* versus *M_v_* tended to be located temporally within 10° of the horizontal meridian (Fig. 3).

Furthermore, likelihood ratio tests were performed to compare the models, which showed similar results as the AIC comparison. *M_v:t_* outperformed *M_t_* in 68 (93%) and *M_v_* in 49 (67%) VF locations. Fifteen (63%) of the 24 VF locations for which no better performance of *M_v:t_* as compared to *M_v_* could be observed using likelihood‐ratio tests were found temporally within 10° of the horizontal meridian (Fig. 3).

### Segmented regression analysis

For a better understanding of the correlation between structural and functional parameters in the course of deteriorating *fVFD*, we performed a segmented regression analysis on a linear mixed model pooling *fCD* or *fRNFL‐T* values of all VF locations, while adjusting for nonindependence of the data points from a single individual. A significant breakpoint was found for both relationships. However, for *fCD*, the breakpoint was detected at a slightly higher *fVFD* compared with *fRNFL‐T* (Table 3). In both SRs, slopes before and after the breakpoint were significant; however, slopes on the right side of the breakpoint were steeper, suggesting a floor‐like behavior left of the breakpoint (Table 3).

**Table 3:** Segmented regression analysis for focal parameters

Parameter	Slope before BP	*fVFD* at BP	Slope change
*fRNFL‐T*	0.65 [0.47, 0.84]	−8.46 [−9.68, −7.25]	0.92 [0.10, 1.73]
*fCD*	0.002 [0.001, 0.003]	−9.43 [−8.38, −10.57]	0.013 [0.009, 0.018]

note: Values are presented with 95% CI in brackets.

BP, breakpoint; *fCD*, focal capillary density; *fRNFL‐T*, focal retinal nerve fiber layer thickness; *fVFD*, focal visual field deviation.

## Discussion

In this study, we investigated the focal SF correlation of OCT‐A‐derived *fCD* and OCT‐derived *fRNFL‐T* with *fVFD* in a dataset of evenly distributed early to advanced POAG. Multivariate model *M_v:t_* was found to be overall superior to univariate models *M_v_* and *M_t_* using AIC and likelihood‐ratio tests. Segmented regression revealed a significant breakpoint for both relationships, *fCD* with *fVFD* and *fRNFL‐T* with *fVFD*; however, the breakpoint occurred at a slightly higher *fVFD* for *fCD*.

To our knowledge, this is the first study of focal SF correlation at the level of single VF test spots in a POAG cohort using a combination of thickness and vascular information. This method allowed us to identify a multivariate regression model using both *fCD* and *fRNFL‐T* as overall superior to the nested univariate models using *fCD* or *fRNFL‐T*. However, we also detected VF regions where the combined model (*M_v:t_*) was not superiorly associated with *fVFD*, compared with the nested model *M_v_*. This was the case for the temporal VF within 10° of the horizontal meridian.

Taking into account the Garway‐Heath model for sectoral division of the peripapillary region, the temporal VF within 10° of the horizontal meridian corresponds to the temporal Garway‐Heath sector. Indeed, using Garway‐Heath sectors for SF analysis, especially temporal peripapillary CD, was previously shown to better correlate with corresponding VF sensitivity than did temporal peripapillary RNFL‐T.^18^ Moreover, Wong *et al*. showed a pronounced floor‐like effect for the inner 10° of the VF, approximately corresponding to the temporal peripapillary Garway‐Heath sector for *fRNFL‐T*, which was lacking for *fCD*.^23^

From an anatomical point of view, the overall superiority of *M_v:t_* is not surprising, as the two parameters *fCD* and *fRNFL‐T* reflect different aspects of RNFL loss and can, therefore, contribute complementarily to a model. While *fCD* is a measure of local presence of perfused capillaries, *fRNFL‐T* represents the local axial RNFL dimension and lacks information on the internal structure of the layer composed of neurons, glia, and vasculature. In this context, the lack of further precision added to model *M_v:t_* by variable *fRNFL‐T* in the temporal VF within 10° of the horizontal meridian could be attributed to the physiologically thinner RNFL‐T in the temporal sector compared with inferior and superior sectors, thus reducing the sectoral dynamic range and, subsequently, minimizing information contributable to the model. Finally, thin RNFL regions are prone to a higher inaccuracy regarding thickness measurements, which could also account for lower associations.

Associations of *fCD* with *fVFD* in *M_v_* were highly significant (*P* < 0.001) at more VF locations than those of *fRNFL‐T* in *M_t_*, and comparison of the models revealed that combined model *M_v:t_* outperformed *M_v_* in fewer VF locations than *M_t_*, suggesting better focal correlation between *fCD* and *fVFD*, compared with *fRNFL‐T* and *fVFD*. Better correlation indices between CD and VFD than between RNFL‐T and VFD were reported by studies investigating these relationships in various glaucoma stages under varying degrees of focality.^9^, ^10^, ^11^, ^12^, ^13^, ^14^, ^15^, ^16^, ^17^, ^18^, ^19^, ^20^, ^21^, ^22^, ^23^ The finding that this also holds true for later glaucoma stages at the level of single VF spots underlines the suitability of OCTA‐based CD measures as structural glaucoma parameters, and it confirms the validity and robustness of using a nerve fiber trajectory–based approach, first applied for SF correlations by Wong *et al*.^23^ in early glaucoma. The reason why *M_v:t_* was not significantly superior to *M_v_*, not only in the temporal VF within 10° of the horizontal meridian (as discussed above) but also in nine additional locations of the superior hemisphere when compared using likelihood‐ratio tests, is currently unknown. However, it can be speculated that minimal *fVFDs* in the superior hemisphere (e.g., by the upper eye lid) not evident and significant in a single SAP session play a role. Assuming that associations of *fRNFL‐T* with *fVFD* are overall weaker than those of *fCD* with *fVFD*, the additional error introduced by minute artifacts at VF spots in the superior periphery produces a higher tendency for rendering the association *fRNFL‐T*:*fVFD* insignificant compared with the association *fCD*:*fVFD*. Similar results concerning peripheral VF locations were found in a focal SF analysis in early glaucoma.^23^

Using SR allowed us to determine BPs at which the respective SF correlation significantly changed. While this was the case for both *fCD* and *fRNFL‐T*, the BP occurred at a nominally, yet not significantly, higher *fVFD* for *fCD* compared with *fRNFL‐T*. While this finding points to an advantageous applicability of CD, as compared with RNFL‐T, for follow‐up at later disease stages, comparative longitudinal studies are required to conclusively answer this question.

Compared with previous studies,^18^, ^29^ the difference we observed in slopes at high VFDs between RNFL‐T and CD was less pronounced. While a definite reason for this is unclear, we speculate that the focal approach, the stage of glaucoma, and the OCT device used to measure RNFL‐T could all play roles. Indeed, it has been shown that RNFL‐T measurements with Spectralis OCT show less pronounced flooring than with other devices.^6^ Finally, caution has to be taken when directly comparing the slopes of CD and RNFL‐T, as they are, on the one hand, differently scaled and, on the other hand, the dynamic ranges and the number of measurement steps are, as previously shown, different.^21^

Our study has several limitations. First, the sample size of 46 patients is relatively small. However, the focal approach and the large number of highly deviated VF spots resulting from the use of a dataset, including one‐third advanced glaucoma patients, can partly compensate for that. Moreover, our study is by design cross‐sectional; therefore, the results are not conceived to be directly applicable to disease progression. As mentioned earlier, longitudinal studies are, therefore, necessary.

In addition, the presence of large vessels within the retinal nerve fiber layer may have affected the performance of RNFL‐T. However, their potential contribution could not be evaluated because current methods of analysis do not allow one to distinguish these components in routine RNFL‐T measurements.

Finally, for the calculation of BPs and slopes, we did not divide the VF based on peripapillary sectors or VF degrees, as done previously.^23^ This was partly to account for the relatively small sample size and partly an effort to obtain reliable point estimates for the parameters obtained from SR analysis.

The main strengths of this study lie in the use of a well‐phenotyped glaucoma cohort evenly distributed in relation to VFD and containing a substantial number (37%) of patients with advanced glaucomatous VF loss who received ophthalmic examinations, OCT, and OCT‐A imaging with the same machine, and SAP at the same clinical setting; these qualities allowed us to apply a VF‐based approach for SF correlation of, at this stage of disease, precise focality.

In conclusion, we present focal SF correlations for CD and RNFL‐T with VFD, using both univariate and multivariate modeling, where the combined use of CD and RNFL‐T was identified to have overall advantageous properties over vascular or thickness measurements alone, with focal exceptions for the former. Using segmented regression modeling, we identified significant changes in the slope for focal CD and RNFL‐T in the course of deteriorating VFD, with a BP for focal CD at higher deviations.

## Author contributions

Conception and design: M.K., B.T., D.S., L.S., G.G., and D.W. Acquisition of data: M.K., N.H., A.S., and D.W. Analysis and interpretation of data: M.K., J.C., B.T., D.S., C.H., O.F., L.S., G.G., and D.W. Drafting of manuscript: M.K. and D.W. Revision of intellectual content of manuscript: N.H., A.S., J.C., B.T., D.S., C.H., O.F., L.S., and G.G. Approval of final version of manuscript: M.K., N.H., A.S., J.C., B.T., D.S., C.H., O.F., L.S., G.G., and D.W. Responsibility for the integrity of the data analysis: D.W.

## Competing interests

The authors declare no competing interests.

### Peer review

The peer review history for this article is available at https://publons.com/publon/10.1111/nyas.14732.

## References

[1] Jonas, J.B. , T. Aung , R.R. Bourne , *et al*. 2017. Glaucoma. Lancet 390: 2183–2193. 28577860 10.1016/S0140-6736(17)31469-1

[2] Stein, J.D. , A.P. Khawaja & J.S. Weizer . 2021. Glaucoma in adults—screening, diagnosis, and management: a review. JAMA 325: 164–174. 33433580 10.1001/jama.2020.21899

[3] de Moraes, C.G. , J.M. Liebmann , F.A. Medeiros , *et al*. 2016. Management of advanced glaucoma: characterization and monitoring. Surv. Ophthalmol. 61: 597–615. 27018149 10.1016/j.survophthal.2016.03.006

[4] Mwanza, J.C. , D.L. Budenz , J.L. Warren , *et al*. 2015. Retinal nerve fibre layer thickness floor and corresponding functional loss in glaucoma. Br. J. Ophthalmol. 99: 732–737. 25492547 10.1136/bjophthalmol-2014-305745 PMC4441828

[5] Hood, D.C. & R.H. Kardon . 2007. A framework for comparing structural and functional measures of glaucomatous damage. Prog. Retin. Eye Res. 26: 688–710. 17889587 10.1016/j.preteyeres.2007.08.001 PMC2110881

[6] Mwanza, J.C. , H.Y. Kim , D.L. Budenz , *et al*. 2015. Residual and dynamic range of retinal nerve fiber layer thickness in glaucoma: comparison of three OCT platforms. Invest. Ophthalmol. Vis. Sci. 56: 6344–6351. 26436887 10.1167/iovs.15-17248 PMC5109982

[7] Bowd, C. , L.M. Zangwill , R.N. Weinreb , *et al*. 2017. Estimating optical coherence tomography structural measurement floors to improve detection of progression in advanced glaucoma. Am. J. Ophthalmol. 175: 37–44. 27914978 10.1016/j.ajo.2016.11.010 PMC5337134

[8] Rao, H.L. , Z.S. Pradhan , M.H. Suh , *et al*. 2020. Optical coherence tomography angiography in glaucoma. J. Glaucoma 29: 312–321. 32053551 10.1097/IJG.0000000000001463 PMC7117982

[9] Liu, L. , Y. Jia , H.L. Takusagawa , *et al*. 2015. Optical coherence tomography angiography of the peripapillary retina in glaucoma. JAMA Ophthalmol. 133: 1045–1052. 26203793 10.1001/jamaophthalmol.2015.2225 PMC4950955

[10] Akagi, T. , Y. Iida , H. Nakanishi , *et al*. 2016. Microvascular density in glaucomatous eyes with hemifield visual field defects: an optical coherence tomography angiography study. Am. J. Ophthalmol. 168: 237–249. 27296492 10.1016/j.ajo.2016.06.009

[11] Scripsema, N.K. , P.M. Garcia , R.D. Bavier , *et al*. 2016. Optical coherence tomography angiography analysis of perfused peripapillary capillaries in primary open‐angle glaucoma and normal‐tension glaucoma. Invest. Ophthalmol. Vis. Sci. 57: OCT611–OCT620. 27742922 10.1167/iovs.15-18945

[12] Yarmohammadi, A. , L.M. Zangwill , A. Diniz‐Filho , *et al*. 2016. Relationship between optical coherence tomography angiography vessel density and severity of visual field loss in glaucoma. Ophthalmology 123: 2498–2508. 27726964 10.1016/j.ophtha.2016.08.041 PMC5362128

[13] Yarmohammadi, A. , L.M. Zangwill , A. Diniz‐Filho , *et al*. 2017. Peripapillary and macular vessel density in patients with glaucoma and single‐hemifield visual field defect. Ophthalmology 124: 709–719. 28196732 10.1016/j.ophtha.2017.01.004 PMC5499385

[14] Mansoori, T. , J. Sivaswamy , J.S. Gamalapati , *et al*. 2017. Radial peripapillary capillary density measurement using optical coherence tomography angiography in early glaucoma. J. Glaucoma 26: 438–443. 28234680 10.1097/IJG.0000000000000649

[15] Geyman, L.S. , R.A. Garg , Y. Suwan , *et al*. 2017. Peripapillary perfused capillary density in primary open‐angle glaucoma across disease stage: an optical coherence tomography angiography study. Br. J. Ophthalmol. 101: 1261–1268. 28148529 10.1136/bjophthalmol-2016-309642

[16] Chen, C.L. , K.D. Bojikian , J.C. Wen , *et al*. 2017. Peripapillary retinal nerve fiber layer vascular microcirculation in eyes with glaucoma and single‐hemifield visual field loss. JAMA Ophthalmol. 135: 461–468. 28358939 10.1001/jamaophthalmol.2017.0261 PMC5847107

[17] Chen, H.S. , C.H. Liu , W.C. Wu , *et al*. 2017. Optical coherence tomography angiography of the superficial microvasculature in the macular and peripapillary areas in glaucomatous and healthy eyes. Invest. Ophthalmol. Vis. Sci. 58: 3637–3645. 28728171 10.1167/iovs.17-21846

[18] Sakaguchi, K. , T. Higashide , S. Udagawa , *et al*. 2017. Comparison of sectoral structure–function relationships in glaucoma: vessel density versus thickness in the peripapillary retinal nerve fiber layer. Invest. Ophthalmol. Vis. Sci. 58: 5251–5262. 29049726 10.1167/iovs.17-21955

[19] Jo, Y.H. , K.R. Sung & S.C. Yun . 2018. The relationship between peripapillary vascular density and visual field sensitivity in primary open‐angle and angle‐closure glaucoma. Invest. Ophthalmol. Vis. Sci. 59: 5862–5867. 30550617 10.1167/iovs.18-25423

[20] Ghahari, E. , C. Bowd , L.M. Zangwill , *et al*. 2019. Association of macular and circumpapillary microvasculature with visual field sensitivity in advanced glaucoma. Am. J. Ophthalmol. 204: 51–61. 30878489 10.1016/j.ajo.2019.03.004 PMC6642677

[21] Moghimi, S. , C. Bowd , L.M. Zangwill , *et al*. 2019. Measurement floors and dynamic ranges of OCT and OCT angiography in glaucoma. Ophthalmology 126: 980–988. 30858023 10.1016/j.ophtha.2019.03.003 PMC6589389

[22] Chen, A. , L. Liu , J. Wang , *et al*. 2020. Measuring glaucomatous focal perfusion loss in the peripapillary retina using OCT angiography. Ophthalmology 127: 484–491. 31899032 10.1016/j.ophtha.2019.10.041 PMC7093216

[23] Wong, D. , J. Chua , E. Lin , *et al*. 2020. Focal structure–function relationships in primary open‐angle glaucoma using OCT and OCT‐A measurements. Invest. Ophthalmol. Vis. Sci. 61: 33. 10.1167/iovs.61.14.33 PMC7774057 33372979

[24] Penteado, R.C. , L.M. Zangwill , F.B. Daga , *et al*. 2018. Optical coherence tomography angiography macular vascular density measurements and the central 10–2 visual field in glaucoma. J. Glaucoma 27: 481–489. 29664832 10.1097/IJG.0000000000000964 PMC5986603

[25] Jansonius, N.M. , J. Nevalainen , B. Selig , *et al*. 2009. A mathematical description of nerve fiber bundle trajectories and their variability in the human retina. Vision Res. 49: 2157–2163. 19539641 10.1016/j.visres.2009.04.029 PMC2848177

[26] Jansonius, N.M. , J. Schiefer , J. Nevalainen , *et al*. 2012. A mathematical model for describing the retinal nerve fiber bundle trajectories in the human eye: average course, variability, and influence of refraction, optic disc size and optic disc position. Exp. Eye Res. 105: 70–78. 23099334 10.1016/j.exer.2012.10.008

[27] Frangi, A.F. , W.J. Niessen , K.L. Vincken , *et al*. 1998. Multiscale vessel enhancement filtering. Presented at Medical Image Computing and Computer‐Assisted Intervention — MICCAI’98.

[28] European Glaucoma Society . 2020. Terminology and Guidelines for Glaucoma. 5th ed. 10.1136/bjophthalmol-2021-egsguidelines 34675001

[29] Rao, H.L. , Z.S. Pradhan , R.N. Weinreb , *et al*. 2017. Relationship of optic nerve structure and function to peripapillary vessel density measurements of optical coherence tomography angiography in glaucoma. J. Glaucoma 26: 548–554. 28333896 10.1097/IJG.0000000000000670

